# Construction of Expression Shuttle Vectors for the Haloarchaeon* Natrinema* sp. J7 Based on Its Chromosomal Origins of Replication

**DOI:** 10.1155/2017/4237079

**Published:** 2017-03-01

**Authors:** Yuchen Wang, Beibei Chen, Linshan Sima, Mengzhuo Cao, Xiangdong Chen

**Affiliations:** ^1^State Key Laboratory of Virology, College of Life Sciences, Wuhan University, Wuhan 430072, China; ^2^Hubei Provincial Cooperative Innovation Center of Industrial Fermentation, Wuhan 430072, China

## Abstract

Haloarchaeon* Natrinema* sp. J7, the first reported archaeon harboring both plasmid and chromosome-based temperate viruses, is a useful model for investigating archaeal virus-host and virus-virus interactions. However, the lack of genetic tools has limited such studies. On the basis of the automatically replicating sequences of the J7 chromosome and the* pyrF* marker, we constructed seven vectors, six of which were confirmed to possess replication ability in a* pyrF-*deletion derivative of J7 (J7-F). Among these vectors, pFJ1, pFJ4, and pFJ6 could be transformed into the host strain with relatively high efficiency (approximately 10^3^ colony-forming units/*μ*g DNA) and were present at about one copy per chromosome. These three vectors could be stably maintained in J7-F without selection and were used for heterologous protein expression. Only pFJ6 was found to be present in the transformed cells in an exclusively episomal, nonintegrated state (one copy per chromosome). In contrast, some pFJ1 and pFJ4 DNA was probably integrated into the J7-F chromosome. In addition, pFJ6 was found to be compatible with pYCJ in J7 cells, suggesting that these two vectors could be used for further studies of virus-virus and virus-host interactions.

## 1. Introduction

Extremely halophilic archaea constitute a group of microorganisms that thrive in hypersaline environments, including solar salterns and natural salt lakes. This group consists of approximately 48 genera and 177 species [1], forming an important part of archaea [2]. Compared to other groups of archaea, these haloarchaea are particularly suitable for research purposes, as they are easily cultured and manipulated in the laboratory. They also serve as useful models for studying archaeal viruses, which exhibit striking morphological diversity and unique gene contents. However, the study of these haloarchaea and their viruses is limited by the availability of appropriate genetic tools. Among the 48 genera of haloarchaea, genetic tools have been developed for only three genera, namely,* Haloferax* [3, 4],* Halobacterium* [5], and* Haloarcula* [6]. Considering that no single archaeal species is representative of the domain as a whole, or even its own specific group, it will be important to develop genetic tools for the many haloarchaea that still lack such resources.

Most haloarchaeal shuttle vectors are derived from endogenous plasmids, such as pGRB1 [7], pHH1 [8], and pNRC100 [9] from* Halobacterium salinarum* or pHK2 [4, 10], pHV2 [11, 12], and pHV1/4 [13] from* Haloferax volcanii*. The replicative function of some shuttle vectors originates from phage elements or chromosomal replication origins (oriCs), such as pUBP1 and pUBP2. These are constructed based on the ΦH replicon [5] and pBBori7, which was created using oriC/orc7 from* Halobacterium* sp. NRC-1 [14]. Most haloarchaea with established genetic tools can be transformed using polyethylene glycol (PEG). However, only a few selectable markers are available for haloarchaea. Novobiocin, which inhibits DNA gyrase (*gyrB*) [15], and mevinolin, which inhibits HMG-CoA reductase [16] are two widely used antibiotics in archaea. Auxotrophic selectable markers involved in amino acid or nucleotide biosynthesis have also been used. These include the* ura3* and* pyrE2* genes involved in uracil biosynthesis [17, 18], the* trpA* gene involved in tryptophan biosynthesis, the* leuB* gene participating in leucine biosynthesis, and the* hdrB* gene used in thymidine biosynthesis [12]. Among these markers,* ura3* and* pyrE2* are particularly useful for gene knockout or replacement studies because they can be counterselected using 5-fluoroorotic acid [18].

The halobacterial genus* Natrinema* gen. nov., was proposed by McGenity et al. based on the phylogenetic analysis of the 16S rRNA gene sequences and taxonomic properties of its member species. Strains in this genus require that the salt concentration be at least 10% to allow for basic cell growth and 19.8% to 25.1% for optimal growth [19]. Seven species have been discovered in this genus so far. Among them is* Natrinema* sp. J7, which was isolated in a Yingcheng salt mine in Hubei province, China. It is the only strain for which genetic manipulation tools had been established [20, 21] and whose genome had been sequenced [22]. Studies of J7 derivatives have focused on the proteases SptA [23, 24] and SptC [25], heat shock protein 70 [26, 27], and DNA fragments conferring promoter activity in the three domains of life. Notably, one of its derivatives, J7-1, was the first archaeon reported to harbor two kinds of temperate haloarchaeal viruses: sphaerolipovirus SNJ1 [28, 29] and pleolipovirus SNJ2 [30]. Accordingly, J7-1 could be used as an excellent model for studying virus-host and virus-virus interactions in archaea. However, the lack of genetic tools hampered these studies.

Previously, integrative plasmids based on the isolated auxotrophic mutants of J7 derivatives were constructed and used to express exogenous proteins in J7 [20]. Recently, a shuttle vector (pYCJ) based on the SNJ1 replicon (nucleotides 1–4481) was also constructed and validated for the expression and purification of heterologous proteins [21]. A number of ORFs and genetic elements controlling virus genome replication, maintenance, and copy number were identified in the SNJ1 replicon. Moreover, the key elements responsible for superinfection exclusion and lytic/lysogenic conversion of the SNJ1 virus are also located in this region (unpublished data). Because any mutation to the SNJ1 replicon would impair the self-replicating ability of the plasmid, the pYCJ vector is not suitable for investigating these regulators and elements. Thus, additional genetic tools must be developed to enable research on these viruses and their host. In this study, seven shuttle vectors derived from the predicted oriCs in the J7 chromosome were constructed. Each vector contained the* pyrF* gene and the autonomously replicating sequence (ARS, one or two predicted oriCs). Six of these vectors (pFJ1, pFJ3, pFJ4, pFJ5, pFJ6, and pFJ7) were successfully transformed into J7-F, but only pFJ1, pFJ4, and pFJ6 could be transformed with relatively high efficiency (10^3^ colony-forming units [cfu]/*μ*g DNA). These three shuttle vectors were studied further and found to be stably maintained at approximately one copy per chromosome in J7-F without selection. pFJ1 and pFJ4 may exist either in the plasmid form or integrated into the host chromosome, whereas pFJ6 replicated only in a nonintegrated state and was compatible with pYCJ in J7 cells. All three vectors were validated for stable expression of the amylase protein in J7-F cells, making them instrumental for further studies on the SNJ1 virus, as well as J7 derivatives.

## 2. Materials and Methods

### 2.1. Strains, Media, and Growth Conditions

Strains used in this study are listed in Table 1. Different derivatives of* Natrinema* sp. J7 were grown in Halo-2 medium at 45°C, as previously described [28, 30]. A J7-F strain lacking the chromosomal* pyrF* gene was a gift from Professor Yuping Huang (College of Life Science, Wuhan University). Minimal medium (MM; 18%) and modified growth medium (MGM; 18%) were prepared as described in the Halohandbook [31].* Escherichia coli* strains DH5*α* and JM110 were used for plasmid DNA construction and were routinely cultured in Luria–Bertani medium at 37°C. Agar (1.5%) was added when solid analogs of the above-mentioned media were needed.

### 2.2. Predicting oriCs in the J7 Chromosome

The web-based tool Ori-Finder 2 was employed to predict oriCs in archaeal genomes [32]. OriCs were predicted based on an integrated analysis of base-composition asymmetry using the Z-curve method, the distribution of origin recognition boxes (ORBs) identified using Find Individual Motif Occurrence [33], and the occurrence of genes that are frequently close to oriCs.

### 2.3. Plasmid Construction

Plasmids and primers used in this study are listed in Tables 1 and 2, respectively. A 0.95 kb* Bam*HI-*Sna*BI fragment consisting of the* pyrF* gene and the 200 bp promoter region of the* fdx* gene (P_*fdx*_) from* Haloferax volcanii* DS2 was cloned from the pNBK-F plasmid into the* Bam*HI and* Sna*BI sites of the pUC-M vector to generate pUC-M-pyrF.

**Figure 1:**
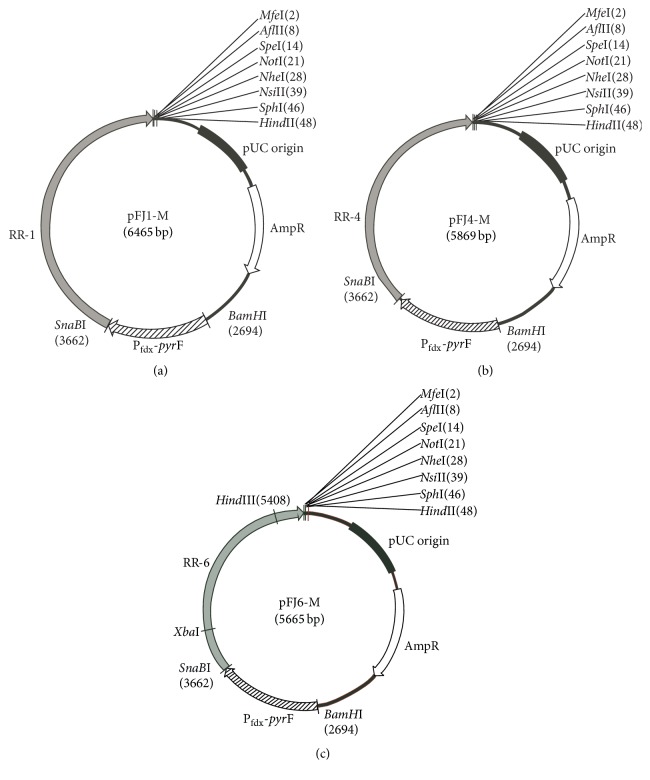
Physical maps of the pFJ1-M (a), pFJ4-M (b), and pFJ6-M (c) plasmids.

Seven* Sna*BI-*Mfe*I-digested ARSs containing one or two predicted oriCs amplified from the J7 genome were ligated into the digested pUC-M-pyrF plasmid to create pFJ1, pFJ2, pFJ3, pFJ4, pFJ5, pFJ6, and pFJ7. The specific locations of the 12 predicted oriCs and seven ARSs are shown in Table 3. A 1.7 kb fragment encompassing* amyH* and its 200 bp promoter (Apro-amyH) was amplified from* Haloarcula hispanica* DSM4426. Restriction enzyme sequences for* Mfe*I,* Afl*II,* Spe*I, and* Not*I were added to the 5′ end of the fragment and* Not*I,* Nhe*I,* Nsi*I, and* Sph*I sites were added to the 3′ end. The* Mfe*I-Apro-amyH-*Sph*I fragment was ligated into pFJ1, pFJ4, and pFJ6 to generate pFJ1-A, pFJ4-A, and pFJ6-A, respectively. After digestion with* Not*I followed by self-ligation, the Apro-amyH fragment was removed from each vector to construct pFJ1-M, pFJ4-M, and pFJ6-M. The physical maps of pFJ1-M, pFJ4-M, and pFJ6-M are shown in Figure 1. A 200 bp fragment encompassing the upstream region of the 3916 gene (3916 pro) located in the J7 chromosome was amplified and ligated into pFJ1-M, pFJ4-M, and pFJ6-M to generate the plasmids pFJ1-M-3916, pFJ4-M-3916, and pFJ6-M-3916, respectively, which were used for plasmid copy number determination.

### 2.4. Transformation Method

Genetic transformation of J7-F cells was performed at room temperature using PEG, as previously described [21]. MM (18%) was prepared and used to select and propagate the* Natrinema* sp. J7-F transformants.

### 2.5. Stability and Maintenance of the Shuttle Vectors

To assess the structural stability of the vectors, we isolated pFJ1, pFJ4, and pFJ6 from J7-F transformants, back-transformed them into* E. coli* for amplification, extracted the plasmids, and subjected them to restriction enzyme digestion. The plasmid DNA was extracted from* E. coli* (without being transformed into J7-F cells), digested, and used as a positive control.

Maintenance of the pFJ1, pFJ4, and pFJ6 shuttle vectors was evaluated by calculating the rate at which each plasmid was lost per generation during nonselective growth, as previously described [21]. Briefly, selected clones were cultured to exponential phase for approximately five days at 45°C in 5 mL 18% MM. The cultures were then diluted 1 : 100 in 5 mL of fresh Halo-2 medium and incubated for 24 h. This dilution process was repeated 12 times (the doubling time of J7 cells was ~2.9 h). Every two dilutions, the cultures were spread onto 18% MGM plates (nonselective condition for J7-F cells). Fifty random clones were selected and reseeded onto 18% MGM or 18% MM plates. The numbers of clones that grew on both kinds of plates were counted. Survival on 18% MM plates indicated maintenance of the vector.

### 2.6. Southern Blot Analysis

Southern blot analysis was performed to determine whether the shuttle vectors (pFJ1, pFJ4, and pFJ6) had integrated into the J7-F chromosome and to measure their copy numbers. J7-F cells transformed with pFJ1, pFJ4, or pFJ6 were cultivated in 200 mL 18% MM and harvested in exponential phase. Their genomic DNA was isolated as previously described [34], digested at the only* Hin*dIII site in the shuttle vectors, and separated by 1% (w/v) agarose gel electrophoresis. After denaturation in alkaline solution (1 M NaCl, 0.5 M NaOH), DNA samples were transferred to a positively charged nylon membrane. Subsequently, the DNA was fixed by incubation at 80°C for 2 h and probed using a* pyrF* fragment labeled with a digoxigenin- (DIG-) dUTP random primer, based on the instructions of the DIG-High Prime DNA Labeling and Detection Starter Kit I (Roche). To determine plasmid copy number, a 200 bp upstream fragment of the J7 3916 gene, present at one copy per chromosome, was amplified and ligated into pFJ1, pFJ4, and pFJ6. Then, total DNA isolated from J7-F/pFJ1-3916pro, J7-F/pFJ4-3916pro, and J7-F/pFJ6-3916pro was digested with* Sau*3AI and subjected to Southern blot analysis as described above, using the DIG-labeled 3916 pro sequence as a probe.

### 2.7. Amylase Activity Assay

Specific amylase activity of the supernatants from CJ7/pYCJ-Apro-amyH, CJ7-F/pFJ1-Apro-amyH, CJ7-F/pFJ4-Apro-amyH, and CJ7-F/pFJ6-Apro-amyH was measured as previously described [21]. One unit of amylase activity was defined as the quantity of amylase required to hydrolyze 1 mg of starch in 1 h.

## 3. Results

### 3.1. Analyzing and Cloning of Replication Regions of the J7 Chromosome

To construct shuttle vectors for J7 derivatives, oriCs in the J7 chromosome were predicted using the web-based Ori-Finder 2 tool. As shown in Table 3(a), 12 oriCs were predicted in the J7 chromosome; most of them had a relatively low GC content compared to that of the J7 chromosome (64%) and all of them contained ORBs. Except for oriC3 and oriC10, the other oriCs were all adjacent to* cdc6* genes, encoding replication-initiating proteins. However, the locations of some oriC pairs, such as oriC1 and oriC2, oriC5 and oriC6, and oriC7 and oriC8, as well as oriC11 and oriC12, were very close and adjacent to the same* cdc6* gene (Table 3(a)). Five presumed ARSs containing one or two oriCs and their adjacent* cdc6* gene were amplified (Table 3(b)). These were then ligated into pUC-M-pyrF to construct the pFJ1, pFJ2, pFJ3, pFJ4, and pFJ5 shuttle vectors. In addition, oriC12 and oriC3, which were not adjacent to the* cdc6* gene, were also amplified and used to construct pFJ6 and pFJ7, respectively. As shown in Table 3(b), all vectors except pFJ2 were successfully transformed into J7-F cells. Transformation efficiency of pFJ1, pFJ4, and pFJ6 was approximately 10^3^ cfu/*μ*g DNA, a little lower than that of the SNJ1 replicon-based vector, pYCJ [21]. Only a few transformants were obtained after transforming JF-7 cells with pFJ3, pFJ5, and pFJ7. These results indicate that at least six regions in the J7 chromosome possess replication ability, which is consistent with the presence of multiple replication origins in the chromosome of most haloarchaeal strains [35].

### 3.2. Maintenance and Structural Stability of the oriC-Based Vectors in J7-F Cells

**Figure 2:**
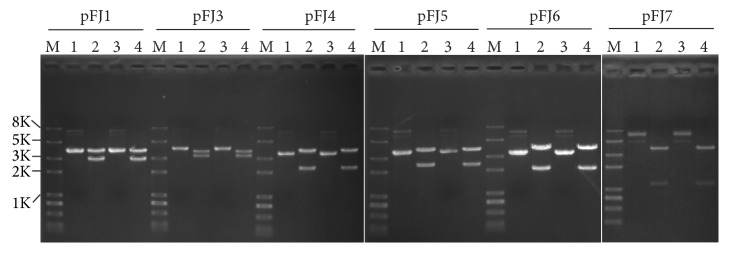
Structural stability of the shuttle vectors in J7-F cells. Pretransformed plasmids (extracted from* E. coli* before being transformed into J7-F, lanes 1 and 2) and back-transformed plasmid (extracted from* E. coli* following transformation into J7-F, lanes 3 and 4) were digested with restriction enzymes to compare their structural stabilities. M, 2 kb plus II marker; lanes 1 and 3, undigested plasmids; lanes 2 and 4, plasmids digested with* Sna*BI and* Mfe*I.

To assess the structural stability of all shuttle vectors (pFJ1, pFJ3, pFJ4, pFJ5, pFJ6, and pFJ7) and verify their ability to replicate independently in J7-F cells, plasmid DNA was extracted from each vector transformant and back-transformed into* E. coli* (posttransformed plasmids), reisolated, and subjected to enzymatic digestion. Shuttle vectors extracted from* E. coli* (pretransformed into J7-F cells) were used as positive controls. As shown in Figure 2, pretransformed (lanes 2) and posttransformed (lanes 4) plasmids showed similar digestion profiles, indicating that structural stability of all constructs was maintained in J7-F cells and that these six vectors replicated independently of the chromosome. As mentioned above, pFJ3, pFJ5, and pFJ7 could be successfully transformed into J7-F cells, but their transformation efficiencies were too low to be suitable for genetic manipulation of J7 cells. Hence, these three vectors were excluded from further studies.

**Figure 3:**
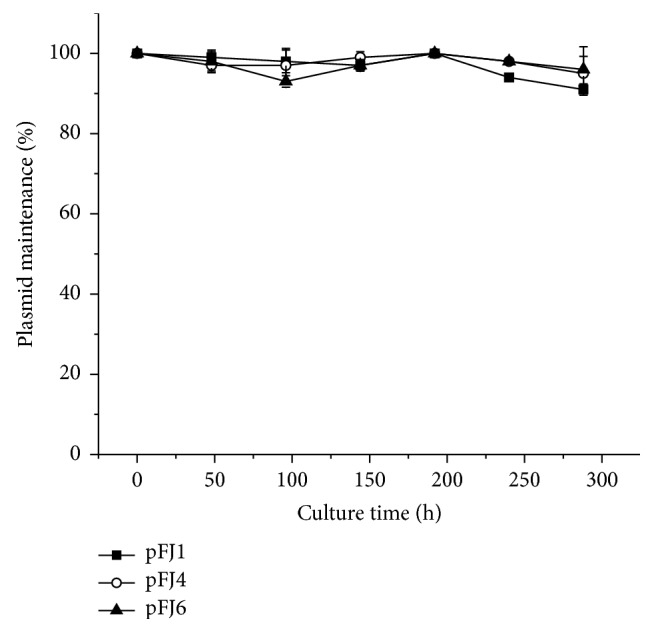
Maintenance of the pFJ1, pFJ4, and pFJ6 shuttle vectors in J7-F cells. J7-F cells containing different shuttle vectors (50 *μ*L) were diluted in 5 mL Halo-2 medium and grown for 24 h. For every two dilutions (12 dilutions in total), aliquots were spread onto Halo-2 plates, and 50 random colonies were selected and spotted onto nonselective 18% MGM plates. Maintenance was measured by calculating the percentage of colonies growing on selective 18% MM plates.

Maintenance of pFJ1, pFJ4, and pFJ6 was determined by calculating the survival frequencies on 18% MM during nonselective growth. As shown in Figure 3, all vectors could be stably maintained in J7-F cells after culturing for 12 days (~100 generations), indicating that all of them could be stably segregated into daughter cells during cell growth.

### 3.3. Presence of pFJ6 in Transformed Cells in an Episomal, Nonintegrated State

**Figure 4:**
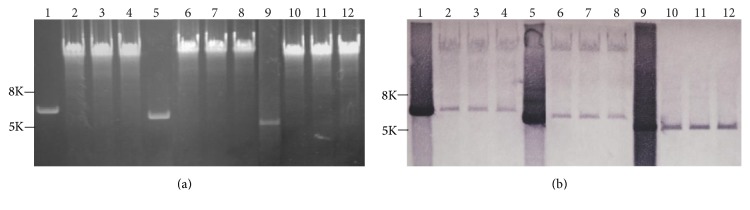
Determination of whether pFJ1, pFJ4, and pFJ6 could integrate into the J7 chromosome by Southern blot analysis.* Hin*dIII-digested total DNA extracted from cultures of J7-F clones carrying the pFJ1 (lanes 2–4), pFJ4 (lanes 6–8), or pFJ6 (lanes 10–12) plasmid were (a) separated on a 1% agarose gel at 20 V for >12 h and then (b) subjected to Southern blot analysis. The pFJ1, pFJ4, and pFJ6 plasmids were propagated in* E. coli*, digested with* Hin*dIII, and loaded on lanes 1, 5, and 9 as controls. The DIG-labeled* pyr*F sequence was used as a hybridization probe.

Southern blot analysis was performed to determine whether pFJ1, pFJ4, and pFJ6 integrated into the J7-F chromosome. Total DNA extracted from three independent J7-F transformants of each plasmid was digested with* Hin*dIII, separated by 1% (w/v) agarose gel electrophoresis (Figure 4(a)), and then subjected to Southern blot analysis. Plasmids extracted from* E. coli* were used as controls and the DIG-labeled* pyrF* gene was used as probe. Only one* Hin*dIII restriction site was found in pFJ1, pFJ4, and pFJ6 plasmids. Thus, digested nonintegrated plasmids from total DNA samples were predicted to be linearized to 5.86 kb, 5.66 kb, and 5.35 kb, respectively, distinguishing them from integrated plasmids. As shown in Figure 4(b), hybridization signals could be observed at every position corresponding to the linearized plasmid DNA, either in the* Hin*dIII-digested DNA samples extracted from* E. coli* (lanes 1, 5, and 9) or in the* Hin*dIII-digested total DNA preparations extracted from cultures of J7-F clones carrying pFJ1 (lanes 2–4), pFJ4 (lanes 6–8), and pFJ6 (lanes 10–12). However, compared with the single hybridization signal obtained using total DNA extracted from pFJ6-transformed J7-F cells (lanes 10–12), the probe also hybridized with a relatively large fragment in the total DNA samples extracted from pFJ1- and pFJ4-transformed J7-F cells (lanes 2–4 and 6–8, resp.). These data indicate that only pFJ6 was present in an episomal, nonintegrated state in J7 cells, whereas pFJ1 and pFJ4 may have integrated into the CJ7-F chromosome. Additional experiments are required to confirm these results.

### 3.4. Relative Copy Numbers of pFJ1, pFJ4, and pFJ6 in J7-F Cells

**Figure 5:**
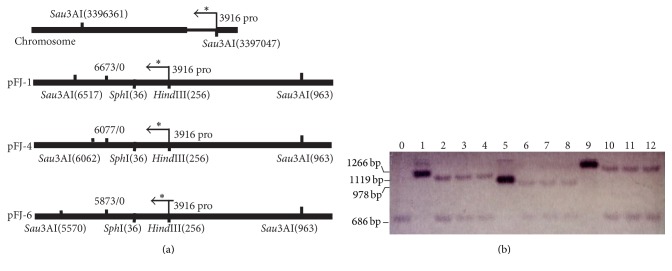
Relative copy numbers of the pFJ1, pFJ4, and pFJ6 plasmids in J7-F cells. (a) Schematic diagrams of the J7 chromosome and indicated vectors. The specific locations of* Sau*3AI sites in the J7 chromosome and plasmids are shown. The 200 bp 3916 pro segment, which was used as a DIG-labeled probe, is indicated with arrows and asterisks. (b) The copy numbers of pFJ1-pro3916, pFJ4-pro3916, and pFJ6-pro3916 were determined by Southern blot analysis.* Sau*3AI-digested total DNA from J7-F cells transformed with pFJ1 (lanes 2–4), pFJ4 (lanes 6–8), or pFJ6 (lanes 10–12) was hybridized with the DIG-labeled 3916 pro sequence. Total DNA samples from J7-F cells (lane 0), pFJ1-pro3916 (lane 1), pFJ4-pro3916 (lane 5), and pFJ6-pro3916 (lane 9) plasmids propagated in* E. coli* were used as controls.

Shuttle vectors containing chromosome oriCs are usually present at one copy per chromosome [36]. We tested whether this was true for pFJ1, pFJ4, and pFJ6. The 200 bp single-copy 3916 pro segment was amplified and ligated into the shuttle vectors used in this study. Total DNA from three randomly selected independent J7-F transformants containing pFJ1-M-3916, pFJ4-M-3916, and pFJ6-M-3916, respectively, was digested with* Sau*3AI, separated by 1% agarose gel electrophoresis, and subjected to Southern blot analysis using a DIG-labeled 3916 pro probe. A 686 bp fragment containing 3916 pro was liberated from the J7 chromosome after digestion with* Sau*3AI, whereas 1119 bp, 978 bp, and 1266 bp fragments containing 3916 pro were generated from pFJ1-M-3916, pFJ4-M-3916, and pFJ6-M-3916 (Figure 5(a)). As shown in Figure 5(b), the DIG-labeled 3916 pro sequence hybridized to the 686 bp fragment from the J7 chromosome and the corresponding fragment of each plasmid. The intensities of the hybridization signals generated from pFJ6-M-3916 and the chromosomal DNA fragment were almost identical, indicating that pFJ6 was present at approximately one copy per chromosome in J7-F cells. In contrast, the intensity of the hybridization signals generated by pFJ1-M-3916 and pFJ4-M-3916 was slightly stronger than that of the corresponding chromosomal DNA fragment, implying that pFJ1-M-3916 and pFJ4-M-3916 were present at more than one copy per chromosome. These results may be attributed to the fact that a portion of pFJ1 and pFJ4 could integrate into the J7 chromosome; however, additional experiments are required to confirm this thesis.

### 3.5. Utility of Shuttle Vectors pFJ1, pFJ4, and pFJ6

**Figure 6:**
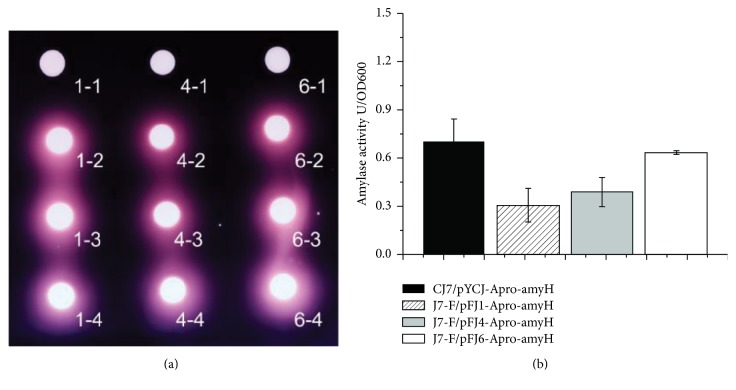
Amylase expression in J7-F cells using pFJ1, pFJ4, and pFJ6. (a) The pFJ1-A, pFJ4-A, and pFJ6-A plasmids were transformed into J7-F cells. Three random transformants were transferred to 5 mL of Halo-2 medium, grown to exponential phase, and 2 *μ*L of each culture was spotted onto 18% MGM plates supplemented with 2% (w/v) soluble starch. After five days, iodine solution was added to the plates, and halos formed immediately around the selected transformants indicating that amylase was successfully expressed. 1-1: J7-F transformed with pFJ1 (negative control); 1-2, 1-3, and 1-4: J7-F transformants harboring pFJ1-A; 4-1: J7-F transformed with pFJ4 (negative control); 4-2, 4-3, and 4-4: J7-F transformants harboring pFJ4-A; 6-1: CJ7 cells transformed with pFJ6 (negative control); 6-2, 6-3, and 6-4: J7-F transformants harboring pFJ6-A (negative control). (b) Amylase-specific activities of supernatants collected from CJ7/pFJ1-Apro-amyH, CJ7/pFJ4-Apro-amyH, CJ7/pFJ6-Apro-amyH, and CJ7/pYCJ-Apro-amyH cultures. One unit of amylase activity was defined as the quantity of amylase required to hydrolyze 1 mg of starch in 1 h.

To determine the utility of these shuttle vectors, the* Haloarcula hispanica* DSM 4426* amy*H gene and its promoter were inserted into pFJ1, pFJ4, and pFJ6. After transforming CJ7 cells with the respective plasmids (pFJ1-A, pFJ4-A, and pFJ6-A), three randomly selected colonies per plasmid were transferred onto 18% MGM plates supplemented with 2% (w/v) soluble starch. As shown in Figure 6(a), transparent halos were detected around the colonies after flooding the plates with iodine solution, suggesting that amylase was well expressed. In contrast, colonies of J7-F cells harboring only pFJ1, pFJ4, or pFJ6 showed no amylase activity, confirming the role of amylase in starch consumption. As shown in Figure 6(b), amylase activity in the supernatants of CJ7-F/pFJ1-Apro-amyH, CJ7-F/pFJ4-Apro-amyH, and CJ7-F/pFJ6-Apro-amyH cultures at the same OD600 was generally lower than that of CJ7/pYCJ-Apro-amyH. This result can be explained by the copy number of pYCJ (one to three copies per chromosome) [21] being higher than that of pFJ1, pFJ4, and pFJ6. The highest amylase activity occurred in CJ7-F/pFJ6-Apro-amyH, suggesting that pFJ6 drove greater protein expression than pFJ1 and pFJ4.

### 3.6. Compatibility of pFJ6 and SNJ1 Replicon-Based pYCJ

Compatible shuttle vectors are excellent tools for investigating protein-protein and protein-DNA interactions in prokaryotes. Previously, we reported the first* Natrinema* sp. J7* E. coli* shuttle vector, pYCJ, which was constructed based on the SNJ1 replicon and validated for stable expression of heterologous proteins. Because pFJ6 could also be maintained in J7 cells and did not integrate into the J7 chromosome, we tested whether pFJ6 and pYCJ were compatible with each other. pFJ6 and pYCJ were cotransformed into J7-F cells and transformants were selected on 18% MM containing 5 *μ*g/mL mevinolin. The presence of pFJ6 and pYCJ in the transformants was detected by PCR using primers targeting* pyrF* and mevR, respectively. The result showed that* pyrF* and mevR were detected in all randomly selected transformants (data not shown), indicating that these two replicons were compatible with each other.

## 4. Discussion

Haloarchaeon* Natrinema* sp. J7 is the first archaeon known to harbor both plasmid- and chromosome-based temperate viruses, SNJ1 and SNJ2. These two viruses display many interesting features. First, the efficient production of SNJ2 could only be achieved in J7 strains coinfected with SNJ1, indicating that SNJ1 promoted the replication of SNJ2 [30]. However, little is known about the mechanism behind this virus-virus interaction. Second, SNJ1 could infect CJ7, which does not harbor pHH205, but could not infect J7-1 (which does harbor pHH205), indicating that the lysogenic SNJ1 virus could establish superinfection exclusion or immunity, a phenomenon poorly understood in archaea. Third, several ORFs and genetic elements controlling virus genome replication, maintenance, and copy number were recently identified in the SNJ1 virus [21]. However, the mechanisms of these genetic elements and genes are completely unknown. All of these observations suggest that J7 and its viruses are excellent models for studying virus-host and virus-virus interactions in archaea. To date, SNJ1 replicon-based pYCJ has been the only shuttle vector enabling genetic manipulation in J7 cells. Because the pYCJ shuttle vector contains the SNJ1 replicon [21], it could not be used for most studies on SNJ1, especially for functional studies on the regulatory proteins encoded by the SNJ1 replicon. In addition, given that only one shuttle vector was available for J7 cells, studies on protein-protein or protein-DNA interactions have also been limited. In this study, seven vectors based on the predicted oriCs in the J7 chromosome and the* pyrF* marker were constructed; six of them could replicate in the uracil auxotrophic J7 strain (J7-F). Three of these plasmids (pFJ1, pFJ4, and pFJ6) could be transformed into J7-F cells with high efficiency (10^3^ cfu/*μ*g DNA). These three vectors were stably maintained in transformed J7-F cells without selection and could be used to express heterologous proteins. Notably, one of these vectors, pFJ6, existed as a plasmid in J7 cells and was compatible with pYCJ. These plasmids should serve as valuable tools for further studies on virus-virus and virus-host interactions in haloarchaea.

The vectors developed in this study displayed many advantages compared to pYCJ. First, they could stably replicate and segregate into daughter cells even under nonselective growth conditions. In contrast, pYCJ has been reported to disappear from CJ7 cells after three days without antibiotic selection [21]. This property is particularly important given that only a few markers are available for archaea. Second, the molecular weights of the vectors constructed here were all around 6 kb, which is much smaller than that of most of the archaeal shuttle vectors previously reported [37], including pYCJ (about 9.9 kb). Consequently, these shuttle vectors should be capable of accommodating larger exogenous fragments and will be much more convenient for genetic manipulation in general. Finally, given their compatibility with SNJ1 (pHH205) and pYCJ, the shuttle vectors constructed in this study and especially pFJ6 could be used for studying the regulators located in the SNJ1 replicon. This will be made possible by expressing only fragments or mutated regions of the SNJ1 replicon. Such kind of molecular manipulation could not be achieved using pYCJ, because any mutation in the 1–4481 region of SNJ1 would impair the stability of pYCJ [21]. In addition, the vectors constructed in this study will provide a useful tool for studying several biological processes in J7 cells. These include superinfection exclusion and lytic/lysogenic conversion of the SNJ1 virus, whose key determinants are located in the 1–4481 region of SNJ1 (unpublished data).

In addition to viral studies, the vectors developed here may be useful for investigating J7 chromosome replication and segregation. Archaeal oriCs normally consist of a long intergenic sequence encompassing an A/T-rich, duplex-unwinding element. They are typically located upstream of a* cdc6*/*orc1* gene, which encodes a putative initiator protein that is homologous to Orc1 of the eukaryotic ORC complex or the helicase loader Cdc6 [38, 39]. Twelve oriCs were predicted in the J7 chromosome using Ori-Finder 2 software; most of them were adjacent to a putative* cdc6* gene. Six of the shuttle vectors containing one or two of the oriCs could replicate independently in J7-F cells, suggesting that at least six of the 12 predicted oriCs had the ability to initiate DNA replication. This is not surprising given that most archaea contain multiple replication origins [38]. However, it is remarkable that the homology between the nucleotide sequences in these six regions and the amino acid sequences of replication initiation proteins is low. It will be of interest to test which oriC in these shuttle vectors is required for replication and whether the adjacent* cdc6* gene is also essential. pFJ6 carrying the predicted oriC10 does not contain a putative* cdc6* gene, but it can replicate, suggesting that some of the* cdc* genes may be responsible for replication initiation at multiple origins. Furthermore, three of the shuttle vectors are maintained at a single copy per chromosome without selection, indicating that their replication is coordinated with that of the chromosome and that they are faithfully segregated into daughter cells. Investigating the molecular mechanisms underlying such coordination will be particularly interesting because the control and coordination of replication initiation at multiple origins in archaea are poorly understood. These vectors are excellent tools for investigating this important question in archaea.

## Figures and Tables

**Table 1:** Strains and plasmids used in this study.

Strain	Description	Source
*Natrinema* sp. J7-1	With SNJ1 proviral genome pHH205, cannot be infected by SNJ1	[28, 34]
*Natrinema* sp. J7-F	Δ*pyrF*, can be infected by SNJ1	Yuping Huang, Wuhan University, Wuhan, China
*Escherichia coli* DH5*α*	*SupE*44 Δ*lacU*169(*φ*80*lac*ZΔM15) *hsdR*17 *recA1 endA1 gyrA96 thi*-1* relA1*	CCTCC
*Escherichia coli *JM110	*dam dcm supE*44 *hsdR*17* thi leu rpsL lacY galK galT ara tonA thr tsx* Δ(*lac*-*pro*AB) F′(*tra*D36 *pro*AB^+^*lacI*^q^* lac*Z ΔM15)	CCTCC
Plasmid		
pNBK-F	AmpR, MevR, used to clone P_fdx_-*pyrF* resistance fragment	Yuping Huang, Wuhan University, Wuhan, China
pUC-M	pUC19 with *Sna*BI, *Mfe*I, *Age*I inserted between *Bam*HI and *Xba*l	Our lab
pUC-M-pyrF	pUC-M with the insertion of 0.95-kb *Bam*HI-*Sna*BI P_fdx_-*pyrF *resistance fragment	This study
pFJ1	pUC-M-pyrF with the insertion of the 2.8-kb *Sna*BI-*Mfe*I predicted chromosomal ARS-1	This study
pFJ2	pUC-M-pyrF with the insertion of the 3.3-kb *Sna*BI-*Mfe*I predicted chromosomal ARS-2	This study
pFJ3	pUC-M-pyrF with the insertion of the 3.2-kb *Sna*BI-*Mfe*I predicted chromosomal r ARS-3	This study
pFJ4	pUC-M-pyrF with the insertion of the 2.2-kb *Sna*BI-*Mfe*I predicted chromosomal ARS-4	This study
pFJ5	pUC-M-pyrF with the insertion of the 2.3-kb *Sna*BI-*Mfe*I predicted chromosomal ARS-5	This study
pFJ6	pUC-M-pyrF with the insertion of the 2.0-kb *Sna*BI-*Mfe*I predicted chromosomal ARS-6	This study
pFJ7	pUC-M-pyrF with the insertion of the 1.1-kb *Sna*BI-*Mfe*I predicted chromosomal ARS-7	This study
pFJ1-Apro-amyH	pFJ1 with the insertion of 1.5-kb *Mfe*I-*Sph*I Apro-amyH fragment containing *Afl*II, *Spe*I, *Not*I, *Nhe*I, and *Nsi*I	This study
pFJ1-M	pFJ1-Apro-amyH digested with *Not*I and then ligated by itself	This study
pFJ4-Apro-amyH	pFJ4 with the insertion of 1.5-kb *Mfe*I-*Sph*I Apro-amyH fragment containing *Afl*II, *Spe*I, *Not*I, *Nhe*I, and *Nsi*I	This study
pFJ4-M	pFJ4-Apro-amyH digested with *Not*I and then ligated by itself	This study
pFJ6-Apro-amyH	pFJ6 with the insertion of 1.5-kb *Mfe*I-*Sph*I Apro-amyH fragment containing *Afl*II, *Spe*I, *Not*I, *Nhe*I, and *Nsi*I	This study
pFJ6-M	pFJ6-Apro-amyH digested with *Not*I and then ligated by itself	This study
pFJ1-M-pro3916	pFJ1-M with the insertion of 200-bp *Nsi*I-*Sph*I pro3916	This study
pFJ4-M-pro3916	pFJ4-M with the insertion of 200-bp *Nsi*I-*Sph*I pro3916	This study
pFJ6-M-pro3916	pFJ6-M with the insertion of 200-bp *Nsi*I-*Sph*I pro3916	This study

**Table 2:** Primers used in this study.

Primers	5′-3′ sequence	Restriction sites
P_fdx_-pyrF-F	AATGGATCCATCTCGGCTTATTCTTTTGATT	*Bam*HI
P_fdx_-pyrF-R	TAATACGTATTATTCTCGATACTGATTGAGTCGCTTC	*Sna*BI
PARS-1-fwd	AATTACGTACGCCCCCGGTGCCTCCTCTCGGA	*Sna*BI
PARS-1-rev	ATTCAATTGACTCGCCGCCGACTACCTCCCCGTCG	*Mfe*I
PARS-2-fwd	AATTACGTATAGCCCGGGAAATACTATCTTTGAGTTCT	*Sna*BI
PARS-2-rev	ATTCAATTGGATCGACGCTGGGATATGAAAAGC	*Mfe*I
PARS-3-fwd	AATTACGTAAACGGCTTTCGGATCGAAAGCAGC	*Sna*BI
PARS-3-rwd	ATTCAATTGTTCGGTCTGCGGTCCCCATTTCC	*Mfe*I
PARS-4-fwd	AATTACGTAGACACACACCACTGTTGCAAGTGAAG	*Sna*BI
PARS-4-rev	ATTCAATTGGTGGCCGCACAAGATCGA	*Mfe*I
PARS-5-fwd	AATTACGTACGATCGTGCCGACGTTACCCGGT	*Sna*BI
PARS-5-rev	ATTCAATTGCGATCCCGAAGACGACCGCGT	*Mfe*I
PARS-6-fwd	AATTACGTAGGAGACGGTCAGAGTTACTGGTCAGT	*Sna*BI
PARS-6-rev	TAACAATTGCAAGGGTTCGTCTGAAACCGTGT	*Mfe*I
PARS-7-fwd	ATATACGTAAGGCTGACTGTATGCGAGT	*Sna*BI
PARS-7-rev	ATACAATTGGCACGACAGTAACAGT	*Mfe*I
3916pro(R)-F	AATATGCATGAGTAAAGTTCGTGTTTCCTTGATTA	*Nsi*I
3916pro(R)-R	ATAGCATGCCGGACAAGACGCCCATTTG	*Sna*BI
Apro-AmyH-M-F	AATCAATTGCTTAAGACTAGTGCGGCCGCGGGAGCCGGAAACGCGGTAGAGATA	*Mfe*I,* Age*I, *Spe*I, *Not*I
Apro-AmyH-M-R	AAGCATGCTATGCATAGCTAGCGCGGCCGCAAGGTAGTGGAAAGCGAGCCAGCGC	*Not*I, *Nsi*I, *Nde*I, *Sph*I
pyrF test-F	CGATCACCGTCAACCCCTACATGG	/
pyrF test-R	TACTGATTGAGTCGCTTCTTCAGTCGTTT	/
MevR test-F	TCGCCTCCCTCGAAGTCGGCACCGT	/
MevR test-R	GAACAACGGCGAAGAAAAGGCAGTCCA	/

**Table:** (a) Characteristic of predicted oriCs

OriCs	Locations	Number of ORB elements	GC content (%)	Adjacent to* cdc6* gene
1	120445–121114	2	51.27%	Y
2	122480–123045	1	60.30%	Y
3	399506–400667	3	56.44%	N
4	434294–435598	8	54.87%	Y
5	1277983–1279633	5	59.37%	Y
6	1280840–1281049	1	66.67%	Y
7	2211660–2211903	2	60.51%	Y
8	2213131–2213664	2	62.89%	Y
9	2573530–2574443	1	55.78%	Y
10	2860326–2861584	7	52.74%	N
11	3151118–3151637	3	60.17%	Y
12	3153862–3154084	1	68.61%	Y

**Table:** (b) Characteristic and replication ability of the shuttle vectors

Shuttle vectors	ARSs	OriCs contained	Chromosomal locations of ARSs (positions)	Containing *cdc6* gene	Transformation efficiency^a^
pFJ1	1	1, 2	120,345–123,145	Y	(1.7 ± 0.6) × 10^3^
pFJ2	2	4	434,194–437,483	Y	0
pFJ3	3	5, 6	1,277,883–1,281,049	Y	(3.9 ± 0.4) × 10^1^
pFJ4	4	7, 8	2,211,560–2,213,764	Y	(2.1 ± 0.1) × 10^3^
pFJ5	5	9	2,572,200–2,574,543	Y	<10
pFJ6	6	10	2,860,000–2,862,000	N	(2.2 ± 0.5) × 10^3^
pFJ7	7	3	399,506–400,667	N	<10

^a^Colony-forming units/*μ*g DNA.
